# Role of Activating Transcription Factor 4 in Metabolic, Neurologic, and Ocular Diseases

**DOI:** 10.3390/cells15060538

**Published:** 2026-03-18

**Authors:** Minwoo Kwon, Anisha Kasi, Stefan Y. Kim, Arya Bairat, Aidan Kumar, Varun Kumar

**Affiliations:** 1 Eye and Vision Research Institute, Department of Ophthalmology, Icahn School of Medicine at Mount Sinai, 1 Gustave L. Levy Pl, New York, NY 10029, USA; minwoo.kwon@icahn.mssm.edu (M.K.);; 2 Department of Pharmacological Sciences, Icahn School of Medicine at Mount Sinai, New York, NY 10029, USA

**Keywords:** ATF4, cellular stress, neurological diseases, metabolic diseases, ocular diseases, cellular signaling

## Abstract

Cells respond to metabolic and environmental challenges through the integrated stress response (ISR), a cellular process that maintains homeostasis under diverse stressors. ATF4 is a key player in this ISR, as it is activated via the PERK–eIF2α–ATF4 pathway. ATF4 induction can elicit adaptive responses, including the regulation of genes involved in metabolism and autophagy, to maintain homeostasis. However, ATF4 activation can also induce apoptosis, leading to a wide spectrum of diseases, including metabolic, neurologic, and ocular pathologies. This duality reflects the highly context-dependent nature of ATF4 signaling. This review aims to synthesize the role of ATF4 in metabolic dysfunction, neurodegenerative diseases, and ocular pathology; the mechanisms underlying its protective versus pathologic effects; and future directions to refine ATF4’s potential as a clinical therapeutic target across different diseases.

## 1. Introduction

Cellular stress responses are essential for maintaining organismal homeostasis in the face of metabolic, environmental, and protein-folding challenges [1]. Among these pathways, the integrated stress response (ISR) occupies a central role by altering mRNA translation and transcriptional programming in response to diverse upstream stressors, including nutrient deprivation, redox imbalance, mitochondrial dysfunction, and endoplasmic reticulum (ER) stress [2]. Activating Transcription Factor 4 (ATF4) is a principal effector of the ISR and functions as a molecular hub that integrates signals from multiple organelles to direct cell fate toward adaptation or apoptosis. Through its induction by eIF2α phosphorylation, ATF4 orchestrates gene networks regulating amino acid metabolism, redox buffering, autophagy, organelle dynamics, and inter-organelle communication [2].

While ATF4 is crucial for adaptive remodeling during acute or physiological stress, accumulating evidence shows that chronic or dysregulated ATF4 activation contributes to metabolic, neurological, and ocular disease [3]. This duality reflects the highly context-dependent nature of ATF4 signaling. The same transcriptional programs that promote survival under nutrient limitation or transient ER stress may become pathogenic under sustained lipotoxicity, environmental injury, or age-related degeneration. Moreover, ATF4 links ER and mitochondrial stress responses with mitochondria-associated ER membranes (MAMs), placing it at an intersection that influences homeostasis, bioenergetics, and cell death pathways [4].

Given ATF4’s broad influence across tissues and its relevance to disease pathogenesis, a deeper understanding of its context-specific roles is essential. In this review, we synthesize current insights into how ATF4 contributes to metabolic dysfunction, neurodegeneration, and ocular pathology, outlining when its signaling is protective versus pathologic and emphasizing common mechanisms (e.g., ER stress, mitochondrial signaling, and MAM remodeling) that link its effects across organ systems. We also discuss the therapeutic implications of modulating ATF4 and identify key gaps and future directions needed to refine its potential as a clinical target. Together, these findings indicate that ATF4 may be therapeutically actionable, yet its manipulation demands particular attention to system-specific factors and the nature of the stress stimulus.

## 2. ATF4 in Metabolic Disease

ATF4 plays a significant role in metabolic regulation, where its activation affects cellular responses to nutrient availability, oxidative stress, and mitochondrial dysfunction. Metabolic organs, including the pancreas, liver, lungs, adipose tissue, skeletal muscle, and cardiovascular system, rely on ATF4-dependent mechanisms to maintain cellular homeostasis under acute stress and fluctuating metabolic demand. However, these same pathways can become maladaptive under chronic nutrient excess, lipotoxicity, or prolonged ER stress, contributing to the development of metabolic disease. The role of ATF4 in metabolic diseases is summarized in Table 1.

### 2.1. ATF4 in Pancreatic β-Cells

In pancreatic β-cells, ATF4 promotes survival during diabetes by upregulating amino acid transporters, antioxidant defenses, and metabolic programs needed to maintain insulin secretion. In the Akita mouse model of diabetes, β-cell-specific deletion of the Atf4 gene accelerates β-cell loss, dedifferentiation, and hyperglycemia, whereas pharmacologic enhancement of the ISR elevates ATF4 expression and preserves insulin production [13]. Additional studies demonstrate that ATF4 is required for sustaining β-cell secretory function during glucose challenge [5]. In contrast, ATF4 becomes detrimental during lipotoxic stress. Fatty acid exposure drives an ATF4-dependent program that accelerates β-cell dysfunction, whereas ATF4-deficient β-cells demonstrate greater resistance to palmitate-induced toxicity [5].

### 2.2. ATF4 in Adipose Tissue and Thermogenesis

ATF4 plays a key adaptive role in brown adipose tissue (BAT), where cold exposure activates an ATF4-dependent thermogenic program. Loss of ATF4 impairs the induction of UCP1, PGC-1α, and other cold-responsive genes, resulting in defective thermogenesis [7]. Beyond canonical UCP1-mediated heat production, ATF4 promotes noncanonical thermogenic pathways, including creatine and calcium cycling, that enhance metabolic flexibility when UCP1 activity is insufficient [14]. However, ATF4’s role is similarly context-dependent. While ATF4 supports cold adaptation, chronic ATF4 activation in BAT during nutrient excess impairs glucose handling. BAT-specific Atf4 deletion improves glucose tolerance and insulin sensitivity despite unchanged body weight and is associated with reduced inflammatory signaling and enhanced glucose transporter expression [10].

### 2.3. ATF4 in the Liver

The liver shows a similar context-dependent shift in ATF4 function. Palmitate induces ATF4-mediated upregulation of CD36, increasing fatty acid uptake and promoting triglyceride accumulation [6]. ATF4 activation also induces cold-inducible RNA-binding protein (CIRP), which stabilizes Angptl3 mRNA. Elevated ANGPTL3 secretion suppresses adipose browning and thermogenic gene expression, thereby reducing systemic energy expenditure and contributing to whole-body metabolic dysfunction [8].

### 2.4. ATF4 in the Cardiovascular System

In the heart, ATF4 supports cardiomyocyte adaptation to pressure overload by activating antioxidant pathways and NADPH-generating metabolic circuits, thereby enhancing redox buffering and limiting fibrotic remodeling. Consistent with this protective role, cardiac-specific ATF4 deletion results in more severe ventricular dysfunction under hemodynamic stress [15]. On the other hand, ATF4 can also contribute to pathological vascular remodeling. Excessive PERK–eIF2α–ATF4 signaling in vascular smooth muscle promotes apoptosis, elastin degradation, and abdominal aortic aneurysm progression, an effect amplified by the upstream epigenetic activator MLL1 [9].

### 2.5. ATF4 in Skeletal Muscle

In skeletal muscle, not all stress adaptations require ATF4. The Lonp1 deletion, for example, activates a mitochondrial unfolded protein response that preserves insulin sensitivity independently of ATF4 [16]. However, ATF4 does participate in ER-mitochondrial communication under specific stress conditions. Loss of the mitochondrial protein OPA1 results in mitochondrial fragmentation and enhanced ER-mitochondrial tethering through an ATF4-dependent mechanism, altering calcium handling and metabolic function [17].

### 2.6. ATF4 in the Lung

In the lung, ATF4 is consistently associated with maladaptive responses. In alveolar epithelial cells, ATF4 activation triggers mitochondrial stress and apoptosis, contributing to pulmonary fibrosis [4]. In fibroblasts, ATF4 promotes serine-glycine biosynthesis, supplying precursors needed for collagen production and extracellular matrix expansion [10]. Pharmacologic inhibition of the ISR with SRIB limits ATF4 translation, restores proteostasis, and attenuates lung fibrosis in experimental models [11]. In COPD, cigarette smoke induces ATF4-CHOP activation in epithelial cells and macrophages, exacerbating apoptosis and inflammation [12].

Across metabolic tissues, ATF4 acts as a highly context-dependent regulator of stress adaptation. Its activation supports β-cell survival, thermogenesis, and myocardial resilience under acute or physiologic stress, yet contributes to lipotoxicity, fibrosis, inflammation, and vascular degeneration under chronic metabolic insults. These contrasting outcomes highlight ATF4’s central role at the intersection of endoplasmic reticulum stress, mitochondrial signaling, and inter-organelle communication. The challenge moving forward will be to selectively utilize ATF4’s adaptive functions while mitigating its pathological effects in metabolic diseases.

## 3. ATF4 in Neurologic Disease

It is well established that the PERK/eIF2α/ATF4/CHOP pathway may be a driver of neurodegenerative diseases. In fact, Galehdar et al. (2010) found that ATF4 induces CHOP expression, which then activates PUMA, a BH3-only member of the Bcl-2 family, which is essential for neuronal cell death of cortical neurons [18]. Given ATF4’s role in neuronal apoptosis, it is pertinent to review the specific pathophysiological mechanisms by which ATF4 is activated in neurological diseases. We review the role of ATF4 in several diseases below, including Parkinson’s Disease (PD), Alzheimer’s Disease (AD), traumatic brain injury (TBI), cerebral ischemia and reperfusion injury (CIRI), and drug-induced neurotoxicity. The role of ATF4 in neurological diseases is summarized in Table 2.

### 3.1. ATF4 in Parkinson’s Disease

ATF4 appears to play an adaptive role in PD, yielding both neuroprotective and pathologic effects depending on the paradigm. ATF4 typically facilitates neuronal apoptosis via the ISR, as evidenced by increased ATF4 expression in substantia nigra neurons in patients with PD relative to controls [19]. However, Sun et al. (2013) found that parkin levels attenuate this effect of ATF4 [19]. When neuronal PC12 cells are exposed to dopaminergic neurotoxins, this typically results in decreased parkin protein levels and cell death via the ISR. However, overexpression of ATF4 partially preserved parkin levels and reduced cell death, suggesting a neuroprotective role for ATF4. In contrast, when parkin was silenced, ATF4 exacerbated neurotoxin-induced cell death. This suggests that ATF4’s role is modulated by the *parkin* gene in PD.

Given that PD neurotoxins typically result in decreases in parkin protein, ATF4 activation in the ISR, and subsequent neuronal apoptosis [19], recent studies have examined the role of ATF4 inhibition in attenuating neuronal cell death and PD progression. For example, in a PD model in Drosophila using pink1 and parkin mutants, Celardo et al. (2016) found that suppressing PERK, the upstream activator of ATF4, conferred neuroprotection by decreasing mitofusin contacts with the ER [20]. Furthermore, this group found that PERK inhibition, via both genetic (deletion of *perk* gene) and pharmacological inhibition with the PERK inhibitor GSK2606414, was neuroprotective in this PD model [20]. Building on this finding, Demmings et al. (2020) found that PD neurotoxins and alpha-synuclein fibrils induced upregulation of ATF4 expression in mouse cortical and mesencephalic dopaminergic neurons, thereby increasing expression of pro-apoptotic factors, Chop, Trb3, and Puma [21]. Furthermore, when this group exposed ATF4-deficient dopaminergic neurons to PD neurotoxins, they observed attenuated neuronal cell death [21]. Furthermore, Aimé et al. (2020) found that the drug adaptaquin reduced expression of ATF4, CHOP, and pro-apoptotic factor Trb3 and preserved parkin levels in response to PD neurotoxins in both cellular and mouse models [22]. Future studies should further explore the clinical effects of the ATF4 inhibitor adaptaquin, given its potential efficacy in PD.

### 3.2. ATF4 in Alzheimer’s Disease

ATF4 has also been found to play an important role in the pathogenesis of AD. Goswami et al. (2023) found that the ER stress proteins, including PERK and ATF4, are upregulated in AD and lead to neurodegeneration and cognitive impairment [23]. When this group inhibited downstream factors of PERK, such as ATF4, they found that neuroinflammation was attenuated, suggesting a pathologic role for ATF4 in AD [23]. Roque et al. (2023) supported this theory, proposing that β-amyloid triggers the heterodimerization of CREB3L2-ATF4 [24]. This hypothesis is supported by their finding that higher levels of CREB3L2 were present in ATF4 coimmunoprecipitates from the dorsolateral prefrontal cortex of patients with AD compared to controls. Furthermore, when this group used RNA sequencing, they identified 53 AD-associated genes directly regulated by CREB3L2-ATF4, indicating that CREB3L2-ATF4 interacts with a substantial subset of the AD transcriptome. Furthermore, CREB3L2-ATF4 activation drives tau hyperphosphorylation, a hallmark of AD pathogenesis. Thus, ATF4 is a key player in the process from β-amyloid production to tau hyperphosphorylation.

Given that ATF4 is a key driver of AD pathogenesis, several studies have examined the effects of ATF4 inhibition on disease progression. For example, in an ApoE4 mouse model of AD, Segev et al. (2015) found that injection of a PKR inhibitor decreased ATF4 expression and rescued memory impairment typical in AD [25]. Likewise, a recent study in an in vitro model of AD found that administration of squalene markedly reduced PERK, ATF4, and CHOP levels, thus attenuating ER stress and decreasing neuronal apoptosis [26]. These two studies suggest that ATF4 plays a harmful role in the pathogenesis of AD and point to ATF4 inhibition as a possible future avenue for the treatment of AD.

ATF4 may also play a protective role in certain paradigms. For example, Pasini et al. (2015) found that ATF4-null mice exhibited greater deficits in long-term memory than controls, suggesting that ATF4 plays a key protective role in synaptic plasticity [27]. Furthermore, in a mouse model of AD, Xiong et al. (2024) found that administering NAD+-boosting agent nicotinamide mononucleotide bolstered the mitochondrial stress response via ATF4 and attenuated mitochondrial stress [28]. This suggests that ATF4 serves an adaptive role in AD, shifting to a protective function in certain paradigms of mitochondrial stress.

### 3.3. ATF4 in TBI

ATF4 has also recently been found to play a role in TBI, a condition in which traumatic injury triggers neuronal necrosis and apoptosis. In a mouse model of severe TBI using a controlled cortical impact device, TBI induced an increase in expression of the PERK/eIF2α/ATF4/CHOP pathway, thereby increasing apoptosis via the ISR [29]. Furthermore, TBI significantly increased ER-mitochondrial coupling at MAMs in the cerebral cortex within the first 24 h post-injury [29]. Interestingly, when ER-mitochondria coupling was reduced by inhibiting PACS2, the inflammatory response mediated by PERK and ATF4 was suppressed, thereby reducing subsequent apoptosis. This suggests that ATF4 may have an important role in regulating MAMs and consequent apoptosis in TBI.

### 3.4. ATF4 in Cerebral Ischemia and Reperfusion Injury (CIRI)

Recent studies suggest that ATF4 plays a key adaptive role in CIRI. In a model of focal cerebral ischemia by middle cerebral artery occlusion in rats, Nakka et al. (2009) found that the PERK-ATF4-CHOP pathway was activated following ischemic injury [30]. In fact, mRNA levels of ATF4 were significantly increased 3–24 h after reperfusion. Furthermore, when Naotaifang formula, a compound shown to be clinically effective for CIRI, was administered to rat models of cerebral ischemia, ATF4 protein levels were reduced [31]. This suggests that ATF4 plays a key role in the injurious effects after cerebral ischemia.

However, ATF4 may also have a protective function in cerebral ischemia. In fact, in an in vitro model of CIRI, silencing ATF4 increased oxidative stress and neuronal apoptosis [32]. In contrast, when ATF4 was upregulated, neurological function improved. Therefore, the exact mechanism underlying the protective role of ATF4 must be elucidated in future studies, as ATF4 may serve as a key therapeutic target for patients with cerebral ischemia.

### 3.5. ATF4 in Neurotoxicity

ATF4 may also be involved in the pathogenesis of neurotoxicity, although the mechanism remains unclear. In a model of Di-(2-ethylhexyl) phthalate (DEHP)-induced neurotoxicity, DEHP elicited downstream ER and mitochondrial stress via PERK, an upstream regulator of ATF4, and mitofusin-2 (Mfn2), a MAM component [33]. The authors suggest that Mfn2 interacts with PERK to stabilize ER-mitochondrial contact, thus leading to mitochondrial dysfunction and neuronal apoptosis [33]. Given that PERK is the upstream regulator of ATF4, ATF4 may also play a role at MAMs in neurodegeneration after toxin exposure. Future studies are necessary to elucidate the role of ATF4, PERK, and Mfn2 in neurodegeneration after toxin exposure.

ATF4 appears to play a significant role in several neurological diseases, including AD, PD, TBI, CIRI, and neurotoxicity. Interestingly, ATF4 plays an adaptive role in many of these diseases, shifting between a detrimental role via the ER stress pathway and a protective role against neuronal apoptosis. Future studies must elucidate the mechanism underlying this shifting role to enable targeted therapies targeting ATF4 in neurological disease.

## 4. ATF4 in Ocular Disease

Beyond metabolic and neurological diseases, recent studies have suggested that ATF4 plays an adaptive role in ocular disease. The summary of ATF4’s role in ocular diseases has been described in Table 3.

### 4.1. ATF4 in Glaucoma

As an ER stress regulator, activated ATF4 promotes apoptosis via the PERK-eIF2α-ATF4-CHOP pathway. In glaucoma, the ATF4-CHOP pathway is activated, leading to trabecular meshwork dysfunction, IOP elevation, and significant structural loss of retinal ganglion cells (RGC) [34,35]. Thus, blocking ATF4 has been found to promote neuroprotection in several animal models by preventing cell death. For example, in a silicone oil-induced ocular hypertension glaucoma mouse model, knockout of CHOP and ATF4 led to preserved RGCs and optic nerve survival [36]. Furthermore, pharmacological inhibition of ATF4 with topical ISRIB, an ATF4/CHOP inhibitor, protected RGCs and produced significant neuroprotective effects in the same glaucoma model [36].

### 4.2. ATF4 in Retinopathy

In a retinopathy model using HPV-16-transformed ARPE-19 retinal pigment epithelial cells, downregulating ATF4 rendered the cells sensitive to oxidative stress [38], suggesting that ATF4 plays a protective role against oxidative stress. This study also further elucidated the mechanism of ATF4’s transcriptional regulation. The researchers found that Nrf2 transcriptionally regulates ATF4, enabling ATF4 to function as a protector against oxidative stress [38].

### 4.3. ATF4 in FECD

Most recently, our lab elucidated the role of ATF4 in Fuch’s Endothelial Corneal Dystrophy (FECD) [37]. We found that ATF4 knockdown after tunicamycin-induced ER stress reduced corneal endothelial cell apoptosis, supporting ATF4 knockdown’s protective function in FECD [37]. ATF4 knockdown also rescued altered mitochondrial bioenergetics and dynamics in FECD [37]. Given ATF4’s diverse roles in ocular disease, further studies are needed to elucidate ATF4’s adaptive functions and the conditions under which its protective and apoptotic roles are activated.

## 5. Critical Gaps for ATF4 Research

### 5.1. Shared and Tissue-Specific Determinants of ATF4 Signaling Across Organ Systems

Although ATF4 has been studied in the context of distinct metabolic, neurologic, and ocular diseases, there are shared mechanistic themes across these systems. Available evidence supports a model in which ATF4 links ER stress, mitochondrial dysfunction, and MAM remodeling, while tissue- and system-specific factors determine its downstream transcriptional output [4,33,37].

One commonality across systems appears to be the duration and magnitude of ISR activation. In pancreatic β-cells, neurons, retinal cells, and cardiomyocytes, transient ATF4 activation promotes adaptive programs that enhance redox buffering, amino acid metabolism, and mitochondrial resilience [5,15,28]. In contrast, sustained or excessive activation often shifts ATF4 signaling toward pro-apoptotic pathways, often mediated through CHOP induction [18,36]. This distinction is observed in metabolic stress, neurodegeneration, glaucoma, and corneal endothelial pathology, suggesting that ISR kinetics may represent a generalizable regulator of ATF4 function.

A second shared feature relates to ER-mitochondrial crosstalk. Across tissues, disruptions in MAM integrity and organelle communication converge on ATF4-dependent transcriptional responses [4,17,33]. Examples include Mfn2-PERK signaling in neurotoxicity, OPA1-dependent ER-mitochondrial tethering in skeletal muscle, mitochondrial unfolded protein responses activation in lung fibrosis, and mitochondrial dysfunction in FECD. These examples support the idea that ATF4 integrates signals from multiple organelles to coordinate adaptation or initiate apoptosis when stress becomes unmanageable.

Transcriptional specificity further determines ATF4 output. Heterodimerization with CHOP drives transcription toward apoptotic pathways, whereas alternative partners such as CREB3L2 in Alzheimer’s disease or Nrf2 in retinal pigment epithelium shift ATF4 activity toward disease-specific regulatory networks [18,24,38]. Emerging evidence also suggests that epigenetic modifiers, such as MLL1 in vascular pathology, may either increase or limit ATF4-driven transcription in a tissue-dependent manner [9]. Therefore, cofactor availability and chromatin context likely represent additional generalizable determinants of ATF4 signaling across systems.

Despite these shared principles, tissue-specific characteristics influence the functional consequences of ATF4 activation. High-energy tissues such as neurons and retinal cells exhibit increased sensitivity to mitochondrial dysfunction, while metabolic tissues respond strongly to nutrient excess and lipotoxic stress. Structural tissues, such as those in the lung and vasculature, show a tendency to drive fibrotic remodeling when ATF4 signaling becomes chronic. These differences suggest that while central determinants of ATF4 activity may be conserved, whether it causes adaptive and pathologic outcomes is dependent on tissue-specific energy demand, stress tolerance, and transcription.

Importantly, the balance between adaptive and maladaptive ATF4 signaling is influenced not only by ISR duration but also by experimental and biological context. In models of acute stress, ATF4 activation is typically linked to protective responses, such as antioxidant defense and mitochondrial stress adaptation [27,28]. However, under chronic or prolonged stress, ATF4 signaling more often promotes CHOP-mediated apoptosis and tissue degeneration [18]. Genetic context further modifies these outcomes, as seen by differences in Parkin status in Parkinson’s disease models or ATF4-null versus wild-type phenotypes in synaptic plasticity studies [19,27]. Additionally, disease stage may impact ATF4 function, with early activation supporting compensation and later activation leading to decompensation and cell loss. These variables likely explain the divergent roles attributed to ATF4 across organ systems.

Together, these findings indicate that ATF4 signaling across metabolic, neurologic, and ocular diseases is shaped partially by generalizable determinants, including ISR duration, metabolic state, organelle coupling, and cofactor availability. Tissue-specific factors ultimately influence the disease phenotype. Understanding how these variables interact is essential for translating ATF4 biology into targeted therapeutic strategies.

### 5.2. Unresolved Questions in ATF4 Biology

**Figure 1:**
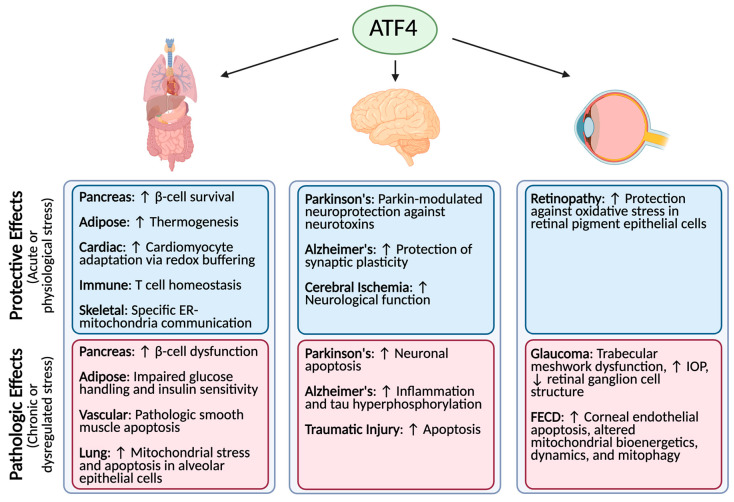
Summary of studies describing the detrimental or protective role of ATF4 in various metabolic, neurological, and ocular diseases.

We present a summary of ATF4’s role in various diseases (Figure 1). Despite significant progress in defining ATF4’s central role in the integrated stress response, several critical knowledge gaps limit our ability to leverage ATF4 biology for targeted therapeutic approaches. A major unresolved question across metabolic, neurological, and ocular disease contexts is what determines whether ATF4 activation drives adaptive or pathologic effects. While the duration and magnitude of ATF4 signaling are often implicated in the literature, the precise thresholds and tissue- and system-specific modifiers that govern this selection are not well defined.

A second critical knowledge gap remains in the context-dependent transcriptional processes regulated by ATF4. Although ATF4 is known to regulate genes involved in amino acid metabolism, redox homeostasis, autophagy, and apoptosis, it remains unclear how its downstream gene networks differ between protective and pathologic states. Emerging evidence suggests that ATF4’s function may be shaped by heterodimerization with other transcription factors (e.g., CREB3L2 in Alzheimer’s disease), but a general framework for understanding ATF4’s transcription across tissues is lacking.

Another gap lies in our understanding of disease-stage-specific effects of ATF4. In several diseases, including Parkinson’s disease, Alzheimer’s disease, diabetes, and ocular pathologies, ATF4 appears to exert different effects depending on metabolic state and disease stage. However, most studies rely on global ATF4 modulation, making it difficult to distinguish between early adaptive responses and later maladaptive effects. Additionally, the role of organelle crosstalk in ATF4 signaling remains incompletely understood. Many studies have implicated ATF4 in regulating ER-mitochondrial communication and MAMs in processes such as neurodegeneration, lung disease, and corneal endothelial pathology. However, whether ATF4 directly influences MAM modeling or acts downstream of altered cross-organelle signaling remains unclear, and how these interactions contribute to metabolic reprogramming versus drive apoptosis remains unresolved.

Finally, there is an important gap in our understanding of the therapeutic modulation of ATF4. While pharmacologic agents targeting the ISR or ATF4 translation show promise in preclinical models, the long-term consequences and safety of modulating ATF4 remain largely unexplored. Given ATF4’s essential role in stress response, inhibition and modulation may carry unintended consequences, underscoring the need to develop precise strategies guided by a deeper mechanistic understanding of ATF4 biology. Overall, gaps highlight the need for integrative, tissue-specific approaches to understand how ATF4 signaling is regulated across tissues, metabolic states, and disease states. Addressing these areas will be essential in our ability to utilize ATF4 as a viable therapeutic target.

### 5.3. Future Directions

As reviewed above, ATF4 plays an adaptive role, often shifting from protective to pathological in various diseases. In metabolic conditions like diabetes, ATF4’s activation can promote β-cell survival, though it can also contribute to lipotoxicity. In neurological diseases like Parkinson’s disease, ATF4 can have protective or deleterious effects on neuronal apoptosis depending on Parkin levels. Lastly, in ocular pathologies such as glaucoma and FECD, ATF4 promotes apoptosis of RGCs and corneal endothelial cells, respectively; however, in retinopathy, ATF4 protects against oxidative stress and the resulting apoptosis. Therefore, it is pertinent for future studies to elucidate the mechanisms underlying the differential activation of ATF4 as a protective versus pathologic effector. We suggest several different avenues for future research below.

A significant consideration in therapeutic development is distinguishing between upstream modulation of the ISR and direct targeting of ATF4. Pharmacologic agents such as PERK inhibitors or ISRIB act upstream by eIF2α phosphorylation and global stress signaling, thereby indirectly influencing ATF4 translation [20,36]. On the other hand, strategies such as ATF4 knockout or inhibition directly suppress ATF4-dependent transcription [37]. These approaches may be substantially different in terms of specificity and systemic impact.

Because ATF4 plays a central role in stress adaptation, mitochondrial homeostasis, and cellular development, prolonged or systemic inhibition may carry unintended consequences. Chronic suppression could impair antioxidant defense, metabolic flexibility, and protective unfolded protein responses in tissues where transient ATF4 activation is beneficial. These considerations suggest that therapeutic strategies should avoid global or sustained suppression. Temporal regulation may be particularly important, as early or transient ATF4 activation can support adaptive responses, whereas sustained activation may promote degeneration. Accordingly, short-term or reversible modulation strategies may offer safer and more disease-relevant approaches.

Tissue-specific targeting is another critical consideration. Local delivery approaches, such as topical administration in ocular disease or organ-directed gene modulation strategies, may reduce systemic exposure and preserve ATF4’s protective roles in other tissues. Also, tissue-specific interventions may allow modulation in vulnerable cell populations (e.g., neurons or corneal endothelial cells) without disrupting global stress adaptation.

Another potential strategy involves pathway-selective targeting. Rather than suppressing ATF4 entirely, selectively targeting downstream pro-apoptotic mediators such as CHOP, or modulating specific ATF4 heterodimer interactions, may preserve beneficial antioxidant and metabolic functions while limiting degenerative signaling [18]. Together, these approaches emphasize precision rather than global suppression as a guiding principle for future therapeutic development.

Building upon these therapeutic principles, several key experimental directions may help clarify how ATF4 can be modulated in a relevant manner. First, ATF4’s multimodal role in disease may be defined by the duration and strength of its activation. If an ATF4 inhibitor, such as the topical ISRIB used by Fang et al. (2023) [36], is administered at titrated concentrations and durations in rodent disease models, this would enable assessment of therapeutic levels of ATF4 activation and resulting clinical effects. Subsequent inferences could then be made about ATF4’s role in disease at various durations and levels of activation.

Second, ATF4’s transcriptome likely shifts between protective and pathologic states. For example, in AD, Roque et al. (2023) found that CREB3L2-ATF4 interacted with a substantial subset of the AD transcriptome [24]. In certain situations where ATF4 acts as a protector, a distinct subset of the AD transcriptome may be activated. By using RNA sequencing to determine the genes associated with ATF4’s differential roles, we can further understand the conditions that precipitate ATF4’s shifting role.

Lastly, given that our lab studies FECD, we set forward our hypothesis that ATF4 plays a key role in the pathogenesis of FECD and therefore could be a key therapeutic target for the disease, which currently has no known cure besides corneal transplant. Our lab recently found that ATF4 knockdown after tunicamycin-induced ER stress decreased corneal endothelial cell apoptosis, suggesting that ATF4 plays a key pathologic role in FECD [37]. However, future studies are necessary to elucidate whether ATF4 activation also plays a protective role in FECD, like it does in other ocular, metabolic, and neurological diseases. This will help further define ATF4’s potential as a therapeutic target in FECD.

Altogether, ATF4’s dual protective and pathologic roles are observed across various metabolic, neurological, and ocular diseases, and further studies are still needed, given its potential clinical utility as a therapeutic target.

## 6. Conclusions

ATF4 is a key component in the integrated stress response and functions as a central regulator of cellular adaptation to metabolic, mitochondrial, and endoplasmic reticulum stress. As summarized in this review, ATF4 contributes to a variety of disease processes across metabolic, neurological, and ocular systems. In metabolic disease, ATF4 can promote adaptive responses such as β-cell survival and thermogenesis but can also contribute to lipotoxicity, fibrosis, and metabolic dysfunction under chronic stress. In neurological disease, ATF4 signaling has been associated with neuronal apoptosis through the PERK–eIF2α–ATF4-CHOP pathway, but it may also support neuroprotective processes depending on the context. In ocular disease, studies have found ATF4 in glaucoma-associated retinal ganglion cell loss and corneal endothelial apoptosis in Fuchs’ endothelial corneal dystrophy, while also demonstrating protective roles in oxidative stress responses.

Despite these advancements, the mechanisms that determine whether ATF4 signaling leads to adaptive or pathological outcomes remain poorly understood. Future studies should focus on better defining the context-dependent transcriptional processes regulated by ATF4 and identifying therapeutic strategies that selectively modulate its activity in specific disease states.

## Figures and Tables

**Table 1:** ATF4’s Role in Metabolic Diseases.

Disease	Key Studies	Main Findings	Ref	ATF4 Role
Pancreatic β-cells dysfunction	Yagan et al., 2025; Griffiths et al., 2023	Chronic lipotoxicity activates ATF4 in pancreatic β-cells, driving fatty acid–induced dysfunction and apoptosis, while ATF4 deficiency confers resistance to palmitate toxicity	[5,6]	Context-dependent
Adipose tissue dysfunction	Lv et al., 2022; Bjorkman et al., 2024	Chronic ATF4 activation in adipose tissue induces CIRP–ANGPTL3 signaling, suppressing adipose browning and thermogenesis, impairing glucose handling, reducing energy expenditure, and promoting systemic insulin resistance during nutrient excess	[7,8]	Pathologic
Abdominal Aortic Aneurysm	Callow et al., 2025	Excessive PERK–eIF2α–ATF4 signaling promotes vascular smooth muscle apoptosis, elastin degradation, and aneurysm progression via epigenetic activator MLL1	[9]	Pathologic
Pulmonary Fibrosis	Jiang et al., 2020; O’Leary et al., 2020; Watanabe et al., 2021	ATF4 activation induces alveolar epithelial mitochondrial stress and apoptosis while enhancing fibroblast serine–glycine metabolism, promoting collagen synthesis, extracellular matrix expansion, and pulmonary fibrosis, which is attenuated by ISR inhibition	[4,10,11]	Pathologic
COPD	Geraghty et al., 2011	Cigarette smoke activates ATF4–CHOP signaling in epithelial cells and macrophages, thereby driving inflammation and apoptosis	[12]	Pathologic

**Table 2:** ATF4’s Role in Neurological Diseases.

Disease	Key Studies	Main Findings	Ref	ATF4 Role
Parkinson’s disease	Sun et al., 2013; Celardo et al., 2016; Demmings et al., 2021; Aimé et al., 2020	ATF4 promotes PD-related apoptosis but can be neuroprotective when parkin is preserved; PERK suppression reduces mitofusin ER contacts, alpha-synuclein increases ATF4 and pro-apoptotic factors, and ATF4 inhibition by adaptaquin decreases dopaminergic cell death.	[19,20,21,22]	Context-dependent
Alzheimer’s disease	Goswami et al., 2023; Roque et al., 2023; Segev et al., 2015; Sarikamis Johnson et al., 2025; Pasini et al., 2015; Xiong et al., 2024	AD activates PERK–ATF4 signaling, which drives neurodegeneration, CREB3L2–ATF4-regulated transcription, and tau hyperphosphorylation, although ATF4 can also support synaptic plasticity. PKR-mediated ATF4 inhibition and squalene-induced suppression of PERK/ATF4/CHOP both reduce ER stress, neuronal apoptosis, and memory impairment in AD models	[23,24,25,26,27,28]	Pathologic
TBI	Chen et al., 2022	TBI activates the PERK/eIF2/ATF4/CHOP pathway and increases MAM coupling, promoting apoptosis, whereas PACS2 inhibition reduces inflammation and neuronal death.	[29]	Pathologic
CI/RI	Nakka et al., 2009; Zhou et al., 2024; Zhao et al., 2025	Cerebral ischemia triggers ATF4–CHOP signaling, though ATF4 upregulation can also lessen oxidative stress and apoptosis	[30,31,32]	Context-dependent
Neurotoxicity	Zhao et al., 2024	PERK–Mfn2-mediated MAM stress induces mitochondrial dysfunction and ATF4-associated neuronal death during DEHP neurotoxicity	[33]	Pathologic

**Table 3:** ATF4’s Role in Ocular Diseases.

Disease	Key Studies	Main Findings	Ref	ATF4 Role
Glaucoma	Mayhew et al., 2025; Kasetti et al., 2020; Fang et al., 2023	Glaucoma-induced activation of PERK–eIF2–ATF4–CHOP promotes trabecular failure, increased IOP, and RGC apoptosis. ATF4/CHOP inhibition and topical ISRIB significantly protect RGCs and the optic nerve.	[34,35,36]	Pathologic
FECD	Qureshi et al., 2026	ATF4 knockdown reduces tunicamycin-induced endothelial apoptosis and rescues mitochondrial bioenergetics and dynamics in FECD.	[37]	Pathologic
Retinopathy	Miyamoto et al., 2011	ATF4 protects the retinal pigment epithelium from oxidative stress, and Nrf2 transcriptionally regulates ATF4. ATF4 downregulation increases oxidative damage and sensitizes cells to stress-induced apoptosis.	[38]	Protective

## Data Availability

No new data were created or analyzed in this study.
